# Pulsed Electro-Magnetic Field (PEMF) Effect on Bone Healing in Animal Models: A Review of Its Efficacy Related to Different Type of Damage

**DOI:** 10.3390/biology11030402

**Published:** 2022-03-05

**Authors:** Mattia Di Bartolomeo, Francesco Cavani, Arrigo Pellacani, Alexis Grande, Roberta Salvatori, Luigi Chiarini, Riccardo Nocini, Alexandre Anesi

**Affiliations:** 1 Unit of Dentistry and Maxillo-Facial Surgery, Surgery, Dentistry, Maternity and Infant Department, University of Verona, P.le L.A. Scuro 10, 37134 Verona, Italy; mattiadiba@hotmail.it (M.D.B.); arrigo.pellacani@libero.it (A.P.); 2 Department of Biomedical, Metabolic and Neural Sciences, Section of Human Morphology, University of Modena and Reggio Emilia, Largo del Pozzo 71, 41125 Modena, Italy; francesco.cavani@unimore.it; 3 Department of Biomedical, Metabolic and Neural Sciences, University of Modena and Reggio Emilia, Via Giuseppe Campi 287, 41125 Modena, Italy; alexis.grande@unimore.it; 4 Department of Medical and Surgical Sciences for Children and Adults, Cranio-Maxillo-Facial Surgery, University of Modena and Reggio Emilia, Largo del Pozzo 71, 41124 Modena, Italy; roberta.salvatori@unimore.it (R.S.); luigi.chiarini@unimore.it (L.C.); 5 Section of Ear Nose and Throat (ENT), Department of Surgical Sciences, Dentistry, Gynecology and Pediatrics, University of Verona, 37129 Verona, Italy; riccardo.nocini@gmail.com

**Keywords:** pulsed, electromagnetic, field, stimulation, PEMF, bone, healing, regeneration, damage

## Abstract

**Simple Summary:**

Pulsed electromagnetic fields (PEMFs) are a type of biophysical stimulation that has been shown to be effective in improving bone regeneration and preventing bone loss. Their use dates back to the 1970s, but a gold standard treatment protocol has not yet been defined. PEMF efficacy relies on the generation of biopotentials, which activate several molecular pathways. There is currently no clear understanding of the effects on bone healing and, in addition, there are several animal models relevant to this issue. Therefore, drawing guidelines and conclusions from the analysis of the studies is difficult. In vivo investigations on PEMF stimulation are reviewed in this paper, focusing on molecular and morphological improvements in bone. Currently, there is little knowledge about the biological mechanism of PEMF and its effect on bone healing. This is due to the variability of crucial characteristics of electro-magnetic fields, such as amplitude and exposure frequency, which may influence the type of biological response. Furthermore, a different responsiveness of cells involved in the bone healing process is documented. Heterogeneous setting parameters and different outcome measures are considered in various animal models. Therefore, achieving comparable results is difficult.

**Abstract:**

Biophysical energies are a versatile tool to stimulate tissues by generating biopotentials. In particular, pulsed electromagnetic field (PEMF) stimulation has intrigued researchers since the 1970s. To date, many investigations have been carried out in vivo, but a gold standard treatment protocol has not yet been defined. The main obstacles are represented by the complex setting of PEMF characteristics, the variety of animal models (including direct and indirect bone damage) and the lack of a complete understanding of the molecular pathways involved. In the present review the main studies about PEMF stimulation in animal models with bone impairment were reviewed. PEMF signal characteristics were investigated, as well as their effect on molecular pathways and osseous morphological features. We believe that this review might be a useful starting point for a prospective study in a clinical setting. Consistent evidence from the literature suggests a potential beneficial role of PEMF in clinical practice. Nevertheless, the wide variability of selected parameters (frequency, duration, and amplitude) and the heterogeneity of applied protocols make it difficult to draw certain conclusions about PEMF effectiveness in clinical implementation to promote bone healing. Deepening the knowledge regarding the most consistent results reported in literature to date, we believe that this review may be a useful starting point to propose standardized experimental guidelines. This might provide a solid base for further controlled trials, to investigate PEMF efficacy in bone damage conditions during routine clinical practice.

## 1. Introduction

Maxillofacial surgery, orthopedics, hand surgery, and neurosurgery are some of multidisciplinary research activities in medicine interested in understanding the mechanism of bone healing and, most of all, if it can be accelerated or improved [1,2,3,4,5,6,7].

The discovery of electrical phenomena related to bone tissue has lead, during the last 50 years, to an exponential increase in both in vitro, in vivo and clinical experimentation, aimed at understanding the application potential of electrical and mechanical energies: to accelerate the healing of fractures, prevent osteoporosis, reduce resorption, accelerate metaphyseal growth, direct differentiation, stimulate cell division etc., [8].

The idea of stimulating bone repair through the application of different types of bio-physical energies (electrical, electromagnetic, mechanical) arises from several experimental observations:

- Bone adapts its shape according to the applied load; this principle is known as Wolff’s law, from the name of the German doctor Julius Wolff who, at the end of the 1800s, described how bone tissue can respond to mechanical load [9].
- It is possible to measure electric potentials on bones in vivo, defined as “biopotentials”, that reflect the metabolic activity of bone itself, these potentials are higher at the metaphyseal level with respect to the diaphyseal one [10,11].
- Bone, when deformed, generates voltage differences due to piezoelectric properties and/or streaming potentials (related to the movement of biological fluids within bone) [8,12,13].
- In case of fracture, a lesion current can be recorded at the fracture site and the whole biopotential distribution of that bone becomes more negative [2].

Taken together, these data indicate that there is a close relationship between the bio-logical activity of bone tissue, mechanical forces (e.g., applied load) and electrical currents. 

Many types of energies have been applied so far in preclinical research in order to understand their interaction with bone. Among the methods used to transmit biophysical energy to biological systems, there is the faradic system, also known as direct current (DC) application through electrodes directly implanted in bone tissue. This system is invasive, increases the risk of infections, requires a surgical intervention and manifested problems related to the electrochemical reactions around electrodes [14]. Therefore, capacitive coupling (CC) systems have been developed, as they are less invasive. They exploit the electrical field generated between two plates placed externally to the limb where a lesion has to be treated [15,16,17]. Mechanical stimulation can be delivered through low intensity pulsed ultra sound (LIPUS). This system is based on the properties of piezoelectric crystals that can generate mechanical waves that are applied to tissues when excited with an alternating current at a certain frequency. The search for the optimal signal characteristics lead to the development of clinical devices approved by the FDA in 1994 [18,19,20]. Nonetheless, recently the role of LIPUS on bone healing has been strongly criticized [21].

Finally, pulsed electromagnetic fields (PEMFs) represent a biophysical stimulation modality that allows the induction of an electric current and a magnetic field in the tissues in a non-invasive way through the application of Helmoltz coils. They can be applied only in a specific area of the body or a total body stimulation can be performed (especially in case of small experimental animal models, like mice or rats).

The search for adequate settings and parameters needed for electromagnetic energy application for rehabilitative purposes in animal model and patients has been underway for many years; nonetheless, a gold standard protocol has not been established at the present moment in pulsed electromagnetic field (PEMF) stimulation. Many in vitro studies showed that electromagnetic stimulation is able to exert different effects on isolated cells, like promoting cell growth and alkaline phosphatase activity on human osteoblast cultures or increasing differentiation markers in precursor cells [22,23,24,25]. Nonetheless, the in vivo environment is much more complex than the in vitro one and allows the investigation of different phases of bone healing from various points of view. Therefore, only in vivo studies in animal models were considered in this narrative review as the basis of the positive effects observed, in particular focusing on those that explored biological pathways (serum and molecular analyses) and morphological variations (histological or micro-CT evaluations), in order to better understand the mechanisms by which electromagnetic stimulation promotes osteogenesis.

The main characteristics of pulsed electromagnetic fields, different forms of bone damage, and the application of rehabilitative electromagnetic field in vivo are discussed in the present review. The aim is to provide clear and concise information on the efficacy of PEMF, in order to lay the foundation for standardized experimental guidelines. This will open the door to studying the effects of PEMF stimulation on bone metabolism as a useful tool in routine clinical practice.

## 2. PEMF Signal Characteristics

The initial research on PEMF stimulation and bone repair, that will be detailed in paragraph 4, was mainly aimed at understanding which parameters of the electromagnetic field would benefit bone healing [26,27,28,29,30]. The approach used in those studies was very similar to pharmacological research; amplitude, frequency, waveform, and exposure length of the biophysical stimuli had to be identified, characterized and optimized. Eventually, a dose response effect using PEMFs was found. Similar to pharmacodynamic studies, this meant that the signal characteristics were fundamental to obtaining a biologically effective response. In order to better understand some technical terms employed in the present review, a brief description of the signal characteristics is presented in this paragraph. The electromagnetic field is generated by Helmoltz coils, single or paired, connected to a generator of continuous electric current [31,32].

**Figure 1:**
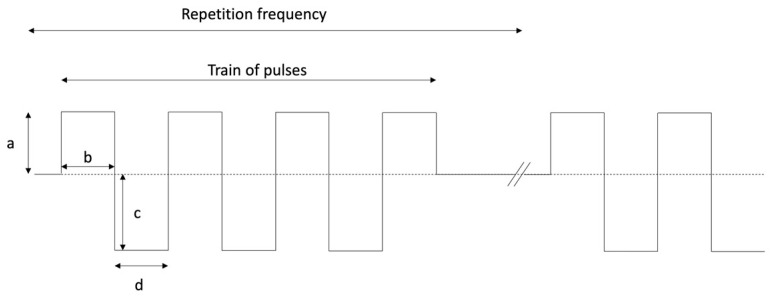
Example of the electric signal generated by a stimulator. a = positive amplitude; b = positive width; c = negative amplitude; d = negative width.

According to the amount and characteristics of the current and of the coils, an electromagnetic field is generated inside the coil or coils. The magnetic field is expressed in Tesla (T) or Gauss (G), where 1G = 10^4^ T, and the electric field in Volts (V) or millivolts (mV). The parameters that characterize PEMFs signals are the repetition frequency (Hertz, Hz) of the trains of pulses generated, the duration and amplitude of each pulse, the symmetry or asymmetry of the pulses and their shape, the duty cycle (the interval between trains of pulses), and the electric and magnetic field generated inside the coil(s). Figure 1 shows an example of the electric signal generated by a stimulator. A review, aimed at comparing PEMF signals of proven efficacy using marketed devices reported in detail the signal characteristics of the most widely used stimulators in clinical studies, that are also present in some articles referenced in this review. The EBI Medical Systems stimulator uses an electric signal characterized by a train of 20 trapezoidal pulses lasting 5 ms that repeat at 15 Hz, giving a peak magnetic field of 1.6 mT during each pulse. The IGEA Medical apparatus produces an induced electric field of trapezoidal shape whose peak is at 0.07 mV/cm at a frequency of 75 Hz and a peak induced magnetic field of 2mT. The ORTHOFIX Inc. stimulator produces a magnetic field signal of 2 mT intensity characterized by trains of pulses with a triangular shape that repeats at 15 Hz [33]. Altogether, this amount of technical information is important for the physician that wants to give informed advice to patients and protect them against non-clinically validated apparatuses that can be found on the market. 

## 3. Animal Models

In the context of studies on the effects of PEMFs on bone healing mechanisms, the main animal model used is murine. The strong prevalence of murine models is probably linked to their accessibility and versatility. Multiple scenarios of damage to the bone can occur both directly (iatrogenic osteotomies) and indirectly (both metabolic and disuse), and all of these are to be considered in studying the biological effect of PEMFs; the murine model is suitable for these perspectives. Rodents are chosen because of many advantages: they are cheap, easily manageable and their bone metabolism is similar to the human one [34]. Moreover, they are small and easily fit in cages to perform PEMF stimulation. Their fast growth rate and accelerated bone metabolism also allow adequate assessment of the efficacy of treatment [35]. In iatrogenic osteotomies both large (such as dogs, sheep, goats, horses and nonhuman primates) and small sized (such as rats, mice and rabbits) animals can be selected. Nonetheless, the use of bigger animals creates difficulties in the management and provision of the treatment proposed [36].

While selected animal species can be similar, there are many differences in the specific models related to the bone impairment conditions.

In the case of postmenopausal osteoporosis, ovariectomized rodents are favored, but also sheep and nonhuman primates can be used. In this scenario, a case-control study can be set up creating a sham-operated group for comparison with the ovariectomized group [37,38]. In all but two of the studies reviewed here, the animal model selected was the ovariectomized female rat, while in the remaining cases ovariectomized mice were used [39,40].

When talking about animal models of bone impairment related to diabetes mellitus, two main categories must be considered: drug-induced and genetically manipulated models. In the first case, streptozotocin and alloxan are the main drugs used in murine and leporine models due to their selective toxicity toward pancreatic β-cells. Regarding mutant strains, many genetic models have been developed over time, with BB Wistar rats, Zucker diabetic fatty rats and db/db leptin deficient mice being the most commonly studied ones [38]. As we can observe, the main models are rodents or rabbits. Although diet-induced diabetic models can be useful in studying the pathogenesis of diabetes mellitus, the long onset interval prevents them from being studied in many osseous damage experiments [38]. 

In the case of steroid-induced osteoporosis, the animal is treated with glucocorticoids (such as dexamethasone, prednisone or methylprednisolone). Oral uptake, subcutaneous or intramuscular injection or continuous intravenous infusion are all routes of administration. Also in this case, the long time period needed to induce bone damage discourages the use of larger sized animals [37,38]. In the present review, rodents were preferably studied, except in one case where New Zealand rabbits were selected [41].

It was observed that many different types of animal models can be used in studying the effect of iatrogenic osteotomies and metabolic impairment. In the setting of disuse osteoporosis, rodents are by far the preferred animal model. As pointed out by Brent et al., rats and mice were used in almost 90% of in vivo experiments regarding disuse-induced bone loss. The techniques chosen to induce immobilization and mechanical unloading were usually tail suspension or hindlimb immobilization (mainly via sciatic neurectomy) [37,42].

## 4. The Dawn of Electro-Stimulation in Bone Healing and the Effects of Different Signal Characteristics

Bassett and collaborators published in Science in 1974, a study performed on dogs in which for the first time they demonstrated the use of pulsed electromagnetic fields to stimulate the repair of a bilateral fibula osteotomy [26]. Only one limb was actively stimulated 24 h a day, while the contralateral acted as a control being equally surrounded by an inactive coil. This study involved stimulation with two fields having different physical characteristics, the former with a 1 ms pulse repeated at a frequency of 1 Hz and a peak voltage induced in the bone of 2 mV/cm, the latter with a 15 µs pulse repeated at a frequency of 65 Hz and a voltage induced peak of 20 mV/cm. After 28 days the treated and control fibulae were removed, radiographed, and subjected to non-destructive mechanical testing and histological analysis. The results of the stimulation were different with the two frequencies used: in particular, the frequency at 65 Hz was effective in accelerating the repair of the osteotomy gap, greatly improving the mechanical resistance of the treated limb versus the control group. This improvement was present both compared to the control group and to the group stimulated at the 1 Hz frequency. The authors rejected the idea that the acceleration effect was linked to thermal phenomena (heating of the tissue due to the Joule effect) as the energies involved were extremely low. It is worth emphasizing that the authors themselves indicated the need to carry out further studies to determine which physical characteristics of the signal were most effective in obtaining the desired biological effect, stating that these characteristics may vary from tissue to tissue. In addition, the clinical use of this technique was suggested to reduce the healing time of fractures, with significant benefits for healthcare facilities in economic terms. A non-negligible aspect of this study was the histological demonstration that stimulation with fields having the aforementioned characteristics did not alter the normal repair process. Moreover, these effects on tissues did not cause an increase in cellularity and mitosis, which could lead to possible oncological degeneration of the cells involved.

A few years later Bassett proposed further work, in line with the previously expressed considerations [27]. The aims were to verify which signal characteristics were optimal in significantly reducing the healing time of a fracture, while evaluating the possibility of making a portable battery powered stimulator. The study was carried out by performing bilateral mid-diaphyseal osteotomies in murine radii. They were divided into 5 groups: one control group and four groups stimulated 12 h a day with signals having different characteristics. Among the various settings used, the one that showed the best results in terms of mechanical resistance and histological aspects had the following characteristics:

○ Duration of the signal: 5 ms;
○ Positive width: 250 µs;
○ Positive amplitude: 17 mV;
○ Negative width: 33 μs;
○ Negative amplitude: 150 mV;
○ Repetition frequency: 5 Hz.

In 1979 De Haas evaluated the effect of electromagnetic fields at different frequencies in an experimental model of rabbit osteotomy [28]. The frequencies used were 0.1, 1 and 4 Hz. The magnetic field intensity was set at 250 Gauss in the first two groups, while it was set at 150 Gauss in the last group. The waveform of the generated signal was square in the first two cases and sinusoidal in the third one. In all groups considered, the stimulation lasted 6 h a day for 5 days a week. The animals were sacrificed 2, 3 and 4 weeks after surgery and the bones were radiographed and dissected for histological analysis. The results showed that the 1 Hz frequency was the most effective in accelerating osteotomy repair. It was true especially during the first two weeks of treatment, when the radiological and histological scores were two times higher in treated groups than in controls. After 4 weeks the effect of the stimulation was not as evident as at 2 weeks and this prompted the authors not to recommend this type of treatment in the clinical field. The authors also verified that the treatment did not involve pathological alterations of the tissues subjected to stimulation.

Afterwards, Pienkowski performed a study to evaluate which signal characteristics were the most effective in stimulating bone repair processes, whether signal asymmetry was an important parameter, and whether an effective signal that consumes little energy could be found [29]. Rabbit fibular osteotomy was used in the experimental model, which had already been used in the scientific literature. The results of the mechanical tests to which the fibulae were subjected 16 days after surgery showed that the asymmetry of the signal is not strictly necessary to improve the mechanical resistance of the osteotomized fibula. In fact, a symmetrical but high intensity signal (400 mV) with a frequency of 15 Hz, 20 μs of signal amplitude and 5ms of pulse amplitude was as effective as an asymmetric signal of similar negative amplitude (450 mV) and the same frequency in doubling the mechanical strength of osteotomized fibulae compared to control fibulae or to fibulae treated with signals of different intensity (45, 120, 640 mV). However, from radiological and histological points of view, there were no significant differences between the treatment groups and the controls.

Subsequently, two years later, the same author published a study to search for the minimum height and width of a rectangular symmetrical pulse that was effective in significantly increasing the stiffness of osteotomized rabbit fibulae [30]. The ultimate aim was again to identify an effective signal that could be generated by small and low-consuming stimulators, easily powered by batteries. Fibular osteotomies were performed on a total of 399 rabbits. The frequency and the width of the pulse trains were kept constant at 15 Hz and 5 ms, respectively. This choice was due to their proven effectiveness and also because their decrease would not have significantly altered energy consumption. The mechanical tests showed that a stimulation with a pulse width of 50 mV was able to significantly increase the mechanical resistance of the treated limb and at the same time reduce the consumption of energy compared to the systems used during the regular clinic practice at that time. An equally significant result was that some voltages (e.g., 10 and 25 mV) did not increase the mechanical resistance of the osteotomized limb compared to controls at all.

To resume, these early studies demonstrated the efficacy of PEMF stimulation on improving bone healing and regeneration.

Nevertheless, there was a great variability both in the parameters used and in the animal models analyzed. Furthermore, in none of these cases were the molecular and morphological markers that will subsequently be the subject of this review, taken into consideration. These markers involve pathways attributable to osteoblastic and osteoclastic activity, angiogenesis patterns and fibroblastic proliferation.

In this regard, it is worth underlining that a number of molecular pathways, with their related signaling molecules and intra-cellular transducers, have been demonstrated to have a capacity to mediate the beneficial effects determined by PEMF on bone regeneration. Examples of such components are represented by bone morphogenetic proteins (BMPs), transforming growth factor-β (TGFβ), mitogen activated protein kinases (MAPKs), phosphatidyl inositol 3 kinase (PI-3K), β-catenin, notch and others. All the listed signals substantially act by favoring bone healing through proliferation, survival and fibrogenic effects exerted on the various cell types involved. These aspects have been previously exhaustively reviewed by experts in the field [43].

Table 1 summarizes the main parameters used to evaluate the effectiveness of PEMF stimulation, together with the abbreviations used.

## 5. Main Results in Different Models

In the following section the effect of PEMF has been mainly studied in relation to direct trauma models and indirect trauma models (such as metabolic damage or disuse damage). It was interesting to notice in the earliest studies regarding osseous healing and PEMF stimulation, the damage was commonly an osteotomic (direct) one. Over the years, the focus has also shifted to metabolic and indirect damage. The following section has been divided into subsections, according to the different damage models studied.

### 5.1. Direct Trauma Models

After these preliminary studies, other researchers tried to explore the effect of PEMF on bone healing in the same animal, treating one limb and using the contralateral as a control. In this way it was possible to exclude inter-animal variability. For example, Canè studied the bone healing in a horse model [44]. The aim of the study was to stimulate bone regeneration in transcortical holes drilled in the third metacarpal bone. The signal characteristics were a repetition rate of 75 Hz, magnetic field of 28 G and an induced electric field of 3.25 mV. The stimulation was applied for 24 h a day. At the two-months checkup after the surgery, an increase in the amount of bone was found inside the holes from 40 to 120% in treated limbs compared to control ones. However, this result was only detectable at the diaphyseal level. Moreover, thanks to the tetracycline double labelling of bone, it was possible to demonstrate that the increase in bone deposition was due to enhanced osteoblast activity. In fact, the mineral apposition rate was almost doubled in treated limbs compared to contralateral ones 30 days after surgery [45]. PEMF treated lesions showed an earlier resorption of the hematoma and higher positivity to ALP during the early stages of bone repair in the same animal model. The reason could lie in an acceleration of the repair process due to the electromagnetic stimulation [46].

Yang applied whole body PEMF stimulation to 7-week old Sprague Dawley male rats that had undergone surgery creating a 8 mm calvarial defect [47]. The defect was filled with collagen sponges soaked in recombinant human bone morphogenetic protein-2 (rhBMP-2) at different concentrations (0, 2.5, 5, 10 μg). Animals were divided into eight groups: (1) control; (2) rhBMP-2 2.5 μg; (3) rhBMP-2 5 μg; (4) rhBMP-2 10 μg; (5) PEMF only; (6) rhBMP-2 2.5 μg + PEMF; (7) rhBMP-2 5 μg + PEMF; (8) rhBMP-2 10 μg + PEMF. The signal characteristics were a pulse width of 12 μs, pulse frequency of 60 Hz and magnetic intensity of 10 G. The stimulation was applied 8 h/day. Rats were sacrificed after four weeks. Micro CT analysis showed that PEMF accelerated bone regeneration, resulting in increased BV and BMD in groups that received 0, 2.5, and 5 μg rhBMP-2.

Midura et al., evaluated the healing of a critical size fibular osteotomy in Sprague Dawley rats [48]. They used the contralateral limb as a control. The stimulation applied was characterized by a frequency of 15 Hz and induced magnetic field of 2 mT (Physio-Stim signal) and it was applied for 3 h/day. After 13–20 days post-surgery, the volume of the bone callus was doubled in treated limbs upon micro CT analysis. When the PEMF stimulation was set at a frequency of 1.5 Hz and an induced magnetic field of 0.02 mT (Osteo-Stim signal), it did not show a significant improvement at 9, 13, 16, 20 days after surgery. Moreover, histological results showed the presence of bone tissue inside the gap in 15 Hz stimulated limbs, mostly fibrocartilage in 1.5 Hz treated limbs, and hyaline cartilage in control limbs. It is clear that different electromagnetic fields exert different effects on bone healing.

Another important experiment was conducted by Liu et al., on 3 month old male Wistar rats [49]. They evaluated the repair mechanisms of osteotomic defect in femurs. The limbs were subjected to PEMF stimulation set at a frequency of 15 Hz for 2 h/day for 7 days. The animals were divided into three groups, differing in the magnetic field intensities of the PEMF stimulation (1 mT, 5 mT and 10 mT). ALP values were higher in all treated groups, while a significant increase in the BMD was only found in animals treated at 5 mT and 10 mT, thus also proving that magnetic field intensity can influence bone deposition differently.

Yonemori drilled a 1 mm hole in the humeral neck of New Zealand rabbits and divided the animals into 5 groups (with/without insertion of Kirshner wire (K-wire), PEMF stimulated/non stimulated, 1 control without K-wire) [50]. The PEMF signal was characterized by a pulse duration of 25 μsec and a frequency of 10 Hz and induced magnetic field of 2 G. The treatment was applied for 12 h/day and lasted for 14 days. The coil was placed around the cage and the animal’s limb was immobilized during the treatment. In the PEMF + K-wire group significantly increased bone deposition and ALP values were detectable with respect to the K-wire only group. Proliferation was higher at 7 and 14 days in the PEMF+K wire group compared to the K-wire only group. PEMF alone was not effective when compared to the pure control, only in presence of K wire. Therefore, the wire influenced the environment and made it responsive to PEMF stimulation.

The only study on humans that had a biopsy of the treated area is the one published by Streit in 2016 [51]. A total of eight subjects who had a fifth metatarsal nonunion fracture were randomly divided into placebo and treatment groups. The BIOMET EBI bone healing system was applied for 10 h/day until clinical success or failure. PEMF consisted of asymmetric 4.5 msec pulses repeated at 15 Hz, with a magnetic field intensity rising from 0 to 12 G in 200 μsec and returning to 0 G in 25 μsec. Biopsies before and after treatment were performed. The expression of placental growth factor (PlGF) was significantly higher in the PEMF-treated group compared to the expression level before PEMF treatment. Other factors trended higher following active PEMF treatment including BDNF and BMP-7 and -5. The time to radiographically detect a bone union was 14.7 weeks on average (range 6 to 21 weeks) in the control group and 8.9 weeks on average (range 6 to 16 weeks) in the PEMF-treated group.

### 5.2. Indirect Trauma Models: Metabolic Damage

The animal models on which to evaluate the effects of PEMF in conditions that metabolically compromise the bone structure are many. The three metabolic conditions most frequently considered are: glucocorticoid-induced osteoporosis (GIOP), diabetes-induced osteoporosis, and ovariectomy-related osteoporosis. The clinical implications of these metabolic alterations, are found in clinical practice daily and therefore have generated a particular interest around them [37,52,53,54,55].

Ovariectomized female mouse models (Sprague-Dawley or Wistar) are one of the most frequently used animal models to understand the impacts of the use of PEMF on bone metabolism. The reason for this popularity is the ease in finding this animal model, and the wide availability of literary data that have used this animal as a hormonal and metabolic model. In fact, osteoporosis in menopause is very common in the population, reaching a prevalence of 10% of women between 60 and 70 years and exceeding 26% in women over 70 years of age. The causes are to be found both in the physiological alteration of bone structures with advancing age, and above all in the hormonal changes typical of the post-menopausal period. The lack of estrogen resulting from the reduction of ovarian hormone activity negatively affects bone turnover, thus determining a predominance of osteoclastic activity. The molecular mechanisms underlying these processes are not yet fully understood, which is why the study of animal models in vivo is a key point of research in this sense. However, other factors can also affect postmenopausal osteoporosis, such as reduced physical activity and obesity [37,56,57,58,59].

The studies that use ovariectomized experimental models are numerous and the settings of the PEMF generators taken into consideration were not very constant. The pulse frequency used varied between 7.5, 8 and 15 Hz, and up to 50 Hz in two studies. The peak intensity of the field was between 0.96 and 3.82 mT. The treatment duration ranged from 4 to 8 weeks, with a daily treatment duration of 40 min to 8 h per day. Despite the great variability in the listed parameters, the results are fairly consistent in highlighting the benefits of stimulation with PEMF on bone metabolism [39,40,60,61,62,63,64,65,66,67].

Zhou (2012) highlighted the role of a possible link between PEMF effects and the Wnt/β-catenin signaling pathway to justify the improvements both from the histomorphometric and biomechanical points of view [62]. Application of PEMF produces variations in the expression levels of some proteins, such as Wnt3a and β-catenin, but also increased gene transcription of Runx2 and the low-density lipoprotein receptor-related protein 5 (LRP5), at the expense of the protein DKK1. Similar results were obtained by Zhu et al. in 2018 where a reduction in the levels of proteins that act in an inhibitory manner on the Wnt/β-catenin signal pathway (in particular DKK1 and Sost), but also a reduction in some genes related to osteoclastic activity (such as TRAP, MMP9, CTSK, TRAF6) [39] were measured. The findings of this work are important because they also highlight that the beneficial effects of stimulation with PEMF are comparable to those of knockout mice for the TNF-α or IL6 genes, suggesting a role of these two mediators in post-menopausal osteoporosis. Similarly, other works have evaluated the consequences of PEMF stimulation in mouse models subjected to ovariectomy. The benefits in terms of BMD, cyto-architectural and biomechanical improvements have been in doubt and are reflected in an increase in biomarkers related to the activation of the Wnt/β-catenin signal pathway. It is interesting to note instead that the role of the RANK-RANKL pathway is not yet completely investigated in relation to stimulation with PEMF.

Mishima, on the other hand, has studied the animal model of ovariectomized mice, by using different methods [66]. Stimulation with PEMF lasted up to 24 weeks and the generator setting was 0.3–1 mT in intensity and 15 Hz in frequency. The results were nevertheless positive, because stimulation with PEMF made it possible to prevent bone loss at the level of the hindlegs.

Controversial results were obtained by Van der Jagt et al. [65]. In their work, in which only architectural parameters evaluated by micro CT were taken into consideration, the beneficial effects of stimulation using PEMF were not evident.

Among others, it is interesting to note the possible role of prostaglandin E2 (PGE2) in stimulating a response in bone, highlighted in Chang’s study [63]. Using PEMF stimulation, a significant improvement in trabecular bone was seen both in terms of bone mass and architectural parameters, with a concomitant reduction in PGE2 values which returned in a short time, to the level of the control group.

The model of diabetic mice is another experimental model useful for studying osteoporosis and bone healing mechanisms. In diabetes it is known how progressive osteoporosis develops as a consequence of the metabolic changes present. Although BMD is not always decreased in patients with diabetes mellitus, bone fragility and the risk of pathological fractures are instead increased. In fact, there is a reduction in bone turnover, due to the alteration of both osteoblastic and osteoclastic activities, and an increase in the production of pro-inflammatory mediators. Hyperglycemia can certainly play a fundamental role in this context, since it blocks the juxtaposition of new bone and at the same time increases the excretion of calcium in the urine. Furthermore, it is believed that protein glycosylation (typical of diabetic patients) can also affect these mechanisms, as well as the structural alteration of collagen. The role of the known alteration of the microvascularization characteristic of diabetes, which plays a role both in terms of bone turnover and in terms of bone healing, is also to be emphasized. There are several animal models that can be used for the study of diabetes, both with gene deficiency (e.g., db/db mice with leptin receptor deficiency) and by induction of diabetes by drug administration (e.g., streptozocin) [52,53,54,55,68].

It can be noted that the use of PEMF provides advantages both in terms of preservation of the microstructure and an increase in bone formation markers, as well as values such as BMD or biomechanical properties in the studies taken into consideration. On the other hand, osteoclastogenesis does not appear to be increased. The PEMF settings used were very variable: the peak intensity of the magnetic field ranged from 2.0 to 3.8 mT, the pulsed burst frequency was 8 Hz or 15 Hz, the total duration of exposure between 8 and 12 weeks and the daily exposure time to PEMF between 40 min and 8 h a day [69,70,71].

GIOP in animal models was evaluated in both mouse and rabbit models. It is induced by high-dose injection of substances such as dexamethasone or metilprednisolone acetate. This condition is therefore linked to the blocking of new bone formation and the increase in bone resorption caused by glucocorticoids, which is particularly accentuated in the first period of drug administration. Molecular pathogenesis seems to be linked both to cytoplasmic receptors for glucocorticoids and to some mediators such as RANK and RANK-L, as well as to the induced systemic hormonal changes (especially related to the reduction of androgens and estrogens) [37,56,72,73].

The results of these experiments are superimposable, as the usefulness of stimulation with PEMF on bone formation has been confirmed, evidenced both as an increase in BMD and in osteo-apposition markers, but also as improvements in terms of the histology and architectural structure of the bone. Among the biomarkers considered in these studies there are some previously mentioned (Wnt, LRP5, β-catenin, OPG, Runx2, DKK1), but also new ones such as PPAR-γ and C/EBP-α. The parameters set were similar to those already described in the other metabolic impairment models (frequency between 8 and 50 Hz, intensity between 1.2 and 4 mT, treatment duration between 4 and 12 weeks, with a daily exposure between 40 min and 4 h) [41,49,74,75,76].

### 5.3. Indirect Trauma Models: Disuse Damage

Disuse osteopenia is a type of indirect bone damage, when bone tissue is subject to a process of involution because it is not subjected to physiological, physical stimulation. Disuse is defined as a reduced activity of the skeleton compared to the usual one. There are no absolute levels of decrease in bone mechanical stimulation that describe disuse osteopenia because it correlates with individual daily bone load. This means that any decrease in regular bone mechanical stimulation can constitute disuse as a stimulus related to reduced exertion. The consequences are reflected in bone metabolism and architecture. Initially, osteoblastic differentiation is reduced, while osteoclastic activity is increased, resulting in overall bone resorption. This phenomenon occurs mainly on the endosteal surface. Subsequently, the loss of bone mineralization density and the deterioration of the trabecular microarchitecture develop as disuse-related effects. Furthermore, disuse is also involved in the alteration of the collagen fibril content and molecular organization [42,77,78].

There are numerous attempts to counteract this degeneration, typically linked to situations of fracture of a limb that require long-term immobilization in order to favor the formation of a correct bone callus. In more recent times, however, this issue has also become a field of interest in aeronautics, with the aim of preventing disuse osteopenia that affects astronauts returning from space missions. As already illustrated at the beginning, with Wolff’s law, the lack of a physiological load on the bone can lead to changes in the bone structure and a reduction in BMD. Furthermore, the reduction of neurological stimulation and changes in the osteoprogenitor cell population also play an important role [37,79,80,81,82].

Stimulation studies with PEMF inherent to this topic have taken into consideration only mouse models, with good results in terms of increased bone apposition. These showed an improvement of histomorphometric parameters (such as Tb.Ar and Tb.N) and also of some markers such as BMD, TGF-β1, OCN and P1NP. Li’s work (2018) is of particular interest since it shows the role of the sAC/cAMP/PKA/CREB signaling pathway in combating disuse osteopenia through its activation, determined by stimulation with PEMF [83,84,85,86].

## 6. Discussion

The effectiveness of PEMF in stimulating bone healing is evident, as can be seen from the results presented. The variability of the parameters set in the various studies is undoubtedly the most critical aspect. In fact, the values of the pulse trains and magnetic field and the treatment modalities (number of hours of treatment per day and number of days of therapy) appear to be varied in scientific literature, as can be seen from Table 2. Nonetheless, the efficacy of treatment with PEMF has been proven in most of the studies considered. Furthermore, this beneficial effect was expressed both in terms of bone healing after the damage and in terms of prevention of the bone damage itself.

Another interesting aspect that emerges from the analysis of the various studies is that scholars initially focused only on direct/osteotomic bone damage. The deepening of bone involvement in metabolic pathologies subsequently took hold, although this is not the most evident aspect when it comes to bone healing. The reason is mainly related to the high prevalence of these pathologies. The most shining example is osteoporosis related to menopause, whose prevention and treatment have assumed a central role in the discourse on womens’ health. Bone damage related to diabetes has also assumed some importance as it is now an almost endemic pathology in the Western world. Its rise is mainly linked to the unhealthy lifestyle and diet adopted in developed countries, but also in some developing countries. GIOP, on the other hand, has risen to the fore due to the great transverse nature of the therapeutic use of high-dose corticosteroids. The studies taken into consideration show that in vivo PEMF stimulation can limit or prevent bone damage in an animal model that resembles the pathological conditions listed above. All this has opened up new scenarios due to the possibility of real clinical implications [87,88,89].

The prevention and treatment of disuse osteopenia are also of great interest and perspective. This is because recent medical advances have allowed an extension of the life expectancy of bedridden patients (both for reasons of age and disabling diseases). The possibility of guaranteeing a better quality of life by preventing bone damage resulting from disuse is therefore clinically important. In addition, possible future space travel is arousing curiosity and among the collateral aspects there is the disuse osteopenia that could ensue. It should therefore be able to be prevented or effectively treated by PEMF stimulation.

However, it is intriguing to note how frequently both molecular and cytoarchitectural parameters are improved after stimulation with PEMF. In particular, the osteoblastic line seems to be the one that benefits most from PEMF stimulation, with a substantial increase in bone deposition and BMD. Although not of strict interest for this review, it should nevertheless be remembered that it has been proven that these improvements have a positive impact on the functional and biomechanical aspects of bone [90,91,92].

Although in vivo animal models are a key step in research into bone healing mechanisms, only studies in daily clinical practice provide the final validation. The limit of the studies analyzed in this review is therefore the lack of clinical data. However, given the current prevalence of both direct bone damage (mainly traumatic) and metabolic or disuse conditions that can affect bone, we believe that new studies on humans in vivo can be conducted, in order to have a more specific picture of the parameter settings and the duration of the sessions to optimize treatment.

In real clinical practice, the efficacy of PEMF stimulation was mainly studied in the field of bone fractures, which are still a global public health problem. Around 178 million new fractures were diagnosed worldwide in 2019, making them one of the most common causes of hospitalization and health care access. Absences from work and reduced productivity, along with disability and reduced quality of life, are significant socioeconomic implications of fractures [93]. This is especially true in complicated fractures that need long-term care, such as cases of delayed union and nonunion.

Therefore, it is evident that any potentially viable intervention that promotes bone healing should be considered for application in routine clinical practice.

Currently, there is controversial data from the literature on the efficacy of PEMFs in implementing the fracture healing process [43].

In a randomized placebo-controlled study conducted in 2012, Hannemann et al., studied the effect of PEMFs in 53 non-surgical acute scaphoid fracture repairs. Clinical and radiological fracture union and functional outcomes were considered as primary and secondary endpoints, respectively. However, no significant differences were found between groups. These results led the authors to not consider PEMF a useful additional tool for accelerating fracture healing compared to immobilization [94].

On the other hand, Martinez and colleagues have suggested that electromagnetic field stimulation can lead to faster bone healing. The authors randomly compared the efficacy of PEMFs versus placebo in promoting consolidation of the surgically treated femoral shaft fracture. A union rate of 75% was found at week 12 in the PEMF group, while in the control group it was 58%. At week 18, bone unions were reached in 94% and 80% respectively. Based on these results, the author concluded that PEMF stimulation might be introduced into clinical practice as an adjuvant tool to accelerate the healing of long bone fractures [95].

In a 2014 meta-analysis, Hannemann et al., systematically reviewed 13 randomized controlled trials that compared PEMF or LIPUS with placebo in stimulating bone growth. Considering the radiological and clinical union time of the fracture as outcome measures, the authors found a significant improvement in radiological healing time in the PEMF treated group. Although the data from the studies reviewed suggested a possible role for PEMF stimulation in the treatment of acute fractures, the authors do not consider them sufficient to recommend this technology in clinical practice [96].

There is also interesting evidence from the literature regarding the application of PEMF in the treatment of complicated fractures. This type of fracture represents a serious clinical problem as well as a major cost to the healthcare system. In a non-randomized setting, Assiotis et al., prospectively examined 52 delayed uninfected consecutive unions and nonunion of the tibial shaft with fracture gaps of less than 1 cm. PEMF stimulation was applied 3 h a day for an average time of 29.5 weeks, with no further restrictions on loading or immobilization. The authors found a success rate of 77.3% suggesting that long periods of PEMF stimulation may increase the likelihood of union [97]. Similarly, Shi et al., observed a union rate of 77.4% after a mean duration of PEMF treatment of 4.8 months. The authors focused on the effect of early application of electromagnetic therapy in delayed joint fractures of long bones 16 weeks to 6 months after surgical treatment. Therefore, their results showed the efficacy of early PEMF stimulation in reducing the pain time of patients with complicated fractures [98].

Indeed, PEMF success rates vary greatly between published studies in the treatment of both acute and delayed or unconsolidated fractures, as well as in other clinical conditions. This is likely due to the different parameter settings used. However, the available data show the possible efficacy of PEMF as a non-invasive, low-cost and safe method to improve bone healing. Further investigations are needed in this regard.

## 7. Conclusions

The versatility of using PEMFs in those situations in which bone metabolism can be compromised appears evident in the light of the results shown. In fact, it has proved useful both in situations where the bone has been directly damaged in a traumatic way and in conditions of bone metabolic impairment (such as osteoporosis induced by diabetes, corticosteroids or ovariectomy), as well as in the prevention of osteoporosis from disuse. In addition, this review provides an effective and concise synthesis of both the parameters set that have proved effective, and of the molecular and morphological variables studied so far in this field.

## Figures and Tables

**Table 1:** Main features studied after PEMF stimulations.

Study Object	Abbreviation Used	Clinical Meaning
Soluble adenylyl cyclase (sAC), cyclic adenosine monophosphate (cAMP), protein kinase A (PKA), and cAMP response element-binding protein (CREB) signaling pathways	sAC/cAMP/PKA/CREB pathway	Pathway promotes bone formation
Wingless-related integration site pathway	Wnt pathway	Pathway promotes bone formation
LDL receptor related protein 5	LRP5	Enhances Wnt pathway activation
Dickkopf1	DKK1	Antagonize Wnt pathway activation
Sclerostin	Sost	
Alkaline phosphatase	ALP	Indirect evaluation of osteoblastic differentiation, proliferation and activity
Collagen type I alpha 1 chain	Col1a1	
Osteocalcin	OCN	
Procollagen type 1 n-terminal propeptide	P1NP	Indirect evaluation of osteoclastic differentiation, proliferation and activity
Cathepsin K	CTSK	
Matrix metalloproteinase 9	MMP9	
Tartrate resistant acic phosphatase	TRAP	
CCAAT/enhancer-binding protein alpha	C/EBP-alpha	
Peroxisome proliferator-activated receptor gamma	PPAR-gamma	
Receptor activator of nuclear factor kappa-Β	RANK	
Receptor activator of nuclear factor kappa-Β ligand	RANKL	
TNF Receptor Associated Factor 6	TRAF-6	Antagonize osteoclastic differentiation and activity
Osteoprotegerin	OPG	
Bone morphogenetic protein-2	BMP-2	Enhance osteoblastic differentiation
Fibroblast growth factor	FGF	
recombinant human Bone Morphogenetic Protein-2	rhBMP-2	
Runt-related transcription factor 2	Runx2	
Transforming Growth Factor Beta 1	TGF-beta 1	
Placental Growth Factor	PlGF	Play a major role in angiogenesis and vasculogenesis, which are key to bone formation
Vascular endothelial growth factor	VEGF	
Angiopoietin-2	Ang	
Brain-derived neurotrophic factor	BDNF	
Tunica interna endothelial cell kinase-2	Tie-2	
Bone Surface/Bone Volume	BS/BV	Morphometric parameters linked to bone formation and evaluable by microCT analysis
Bone Mineral Density	BMD	
Bone Volume	BV	
Bone Volume/Total Volume	BV/TV	
Connectivity density	Conn.D	
Mean trabecular thickness	MTT	
Structure model index	SMI	
Trabecular area	Tb.Ar	
Trabecular number	Tb.N	
Trabecular separation	Tb.Sp	
Trabecular thickness	Tb.Th	

**Table 2:** Range of parameter settings proven efficient in PEMF stimulation, divided according to different bone damage models studied.

	Magnetic Field (Range)	Frequency of the Trains of Pulses (Range)	Duration of Each Session (Range)	Overall Treatment Duration (Range)
Osteotomic damage	0.2–2.8 mT	10–75 Hz	2 h–24 h	2–21 weeks
Ovariectomy induced osteoporosis	0.96–3.82 mT	7.5–50 Hz	40 m–8 h	4–8 weeks
Glucocorticoid induced osteoporosis	1.2–4 mT	8–50 Hz	40 m–4 h	4–12 weeks
Diabetes induced osteopenia	2–3.8 mT	8–15 Hz	40 m–8 h	8–12 weeks
Disuse osteopenia	0.6–3.8 mT	10–50 Hz	40 m–8 h	1–12 weeks

## Data Availability

Not applicable.
